# A pilot randomised control trial of the efficacy of stability-based training with visualisation for people with chronic ankle instability

**DOI:** 10.1007/s11517-022-02533-z

**Published:** 2022-03-05

**Authors:** L. Forsyth, J. Bonacci, C. Childs

**Affiliations:** 1 Faculty of Biomedical Engineering, University of Strathclyde, Glasgow, UK; 2 Centre for Sports Research, School of Exercise and Nutrition Science, Deakin University, Geelong, Australia

**Keywords:** Stability, Virtual reality, Feedback, Chronic ankle instability, Rehabilitation

## Abstract

Chronic ankle instability (CAI) is associated with recurring symptoms that inhibit daily activity. Stability-based rehabilitative training is recommended for CAI. Visualisation (VIS) produces real-time feedback using motion capture and virtual reality. This pilot study aimed to determine the feasibility, adherence, safety, and efficacy of incorporating VIS into stability training for people with CAI. Efficacy was examined through effect of VIS training on dynamic stability, perception of stability, and rehabilitative experience. Individuals with CAI completed a 4-week stability-based training programme with VIS, or without visualisation (NO-VIS). Participants completed the Star Excursion Balance Test (SEBT) and Cumberland Ankle Instability Tool (CAIT) prior to, and after training. Enjoyment of training was recorded using the Physical Activity Enjoyment Scale (PACES-8). Of 17 participants (VIS = 10, NO-VIS = 7), there were 2 drop outs (VIS = 1, NO-VIS = 1). No adverse events were reported, and participant drop-out was due to injury unrelated to the study. The VIS group showed a significantly greater increase in average SEBT reach distance (*d* = 1.7, *p* = 0.02). No significant differences were reported for the CAIT or PACES-8. This study supports the feasibility and safety of stability-based training with VIS in those with CAI. The enhanced performance outcome on the SEBT suggests VIS may enhance stability-based training.

**Graphical abstract:**

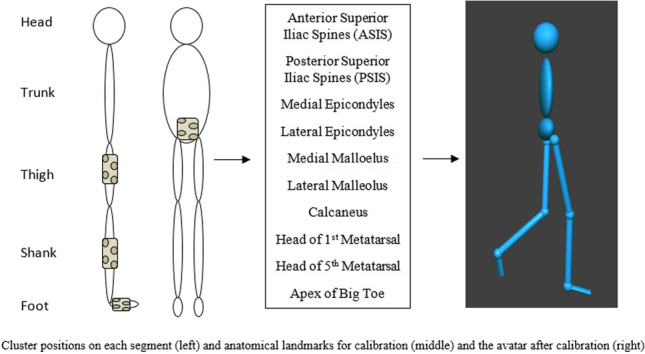

## Introduction

Chronic ankle instability (CAI) is a complicated multi-faceted clinical condition affecting 20–70% of those who have experienced an ankle sprain [1, 2]. CAI is associated with recurrent ankle sprains, mechanical laxity, and/or perceived instability that inhibits daily activity and impacts quality of life [2]. CAI alters joint contact stress and distribution of loading of the articular surface [1, 3, 4]. A link between CAI and ankle post-traumatic osteoarthritis (PTOA) has been established [5]. The degree of physical impairment associated with ankle osteoarthritis is equivalent to that of end-stage kidney disease and congestive heart failure [6]. Surgical treatment includes ankle arthroplasty or arthrodesis [3]. However, fusion of the joint reduces motion, altering stress on other joints. Successful outcomes for arthroplasty are low with a 42% revision rate and lower patient satisfaction [7, 8]. Given the current ankle PTOA treatment options, and that it is a younger population that are typically affected, emphasis should be placed on prevention and conservative management of CAI [3, 6].

Stability-based rehabilitative training is the most recommended rehabilitation strategy for people with CAI [4, 9]. Stability training should challenge the sensorimotor system under different task and environment conditions for optimal rehabilitation outcomes [10]. Examples include reducing the base of support, performing dynamic movements, disturbing centre of gravity, and cognitive and sensory manipulation [11–13].

Virtual reality (VR) presents an enhanced opportunity for interactive simulation using computer software to provide feedback of movement and performance. For stability-based training, this facilitates practice with externally focussed augmented feedback incorporating motor and cognitive manipulation in a safe environment [10, 14, 15]. VR interventions can also easily be adapted and individualised to the needs of the user [16]. A systematic review reported VR balance training to be equally as effective as traditional balance training for prevention and rehabilitation of musculoskeletal lower limb impairments [16]. No study has compared VR balance training to traditional balance training specifically for people with CAI.

VR training enhances stimulation and engagement and has been associated with greater satisfaction and enjoyment of training [17–19]. For those with musculoskeletal impairments, this has increased adherence to rehabilitation [20]. This may create more conducive conditions for rehabilitation since poor programme compliance can hinder a programme’s effectiveness [4, 21]. Compliance with exercise programs has been reported to be as low as 50%, with reduced compliance over time [21, 22]. Low adherence to rehabilitation can be due to low interest or enjoyment in the exercises, negative emotional states, or perceived lack of value [23–25]. VR could be used to create an environment to motivate, monitor, and encourage compliance, thereby leading to more effective rehabilitation.

Visualisation is an emerging technique that connects biomechanical analysis and VR. Visualisation produces real-time feedback by accurately monitoring movement and progress in a diverse, challenging, and controllable environment, representative of real-world situations. Communication could therefore be improved between patient and specialist by making tasks easier to understand, promoting ownership of rehabilitation and intrinsic motivation, whilst enabling objective monitoring of progress [26].

The aim of this pilot study was to determine the feasibility, adherence, safety, and efficacy of incorporating visualisation into stability training for people with CAI. Efficacy was examined through the effect of visualisation on dynamic stability, perception of stability, and rehabilitative experience.

## Methods

### Experimental design

A pilot randomised-controlled trial was conducted to assess the feasibility of a stability-based training programme using visualisation for people with CAI. The study was approved by the University of Strathclyde and Deakin University ethics committee (DEC 2018.243) and received NHS R&D approval for testing on an NHS site (IRAS project ID 247615).

### Participants

Volunteers from local universities and surrounding communities were recruited via poster and social media advertisements and screened for CAI using the International Ankle Consortium guidelines [27]. Participants were required to have experienced all of the following: (1) history of at least one significant ankle sprain, (2) most recent injury to have occurred more than 3 months prior to study participation, (3) history of previous episodes of ‘giving way’, recurrent sprain, or ‘feelings of instability’, and (4) perceived ankle instability determined by a score of < 24 on the Cumberland Ankle Instability Tool (CAIT). The CAIT is a recognised tool for identifying CAI (intraclass correlation coefficient [ICC]_2,1_ = 0.96) [28]. The CAIT contains nine items with a maximum score of 30. A lower score indicates a decreased perception of stability. A history of surgeries or fractures of either lower extremity meant participants were not eligible for participation. All participants provided written informed consent.

### Instrumentation and protocol

All testing and training sessions were supervised and completed in one of three laboratories. The three sites used were (i) Human Performance Laboratory at Glasgow Royal Infirmary, Scotland, UK; (ii) SportScotland Institute of Sport, Scotland, UK; and (iii) Biomechanics laboratory at Deakin University, Geelong, Australia. Testing sessions were conducted the week prior to, and the week following completion of the training block. The training programme was completed biweekly over a 4-week period with visualisation (VIS) or training without visualisation (NO-VIS).

At the pre-training testing session, participant’s age, mass, leg dominance, physical activity levels, sport participation, and ankle injury history were collected.

**Fig. 1:**
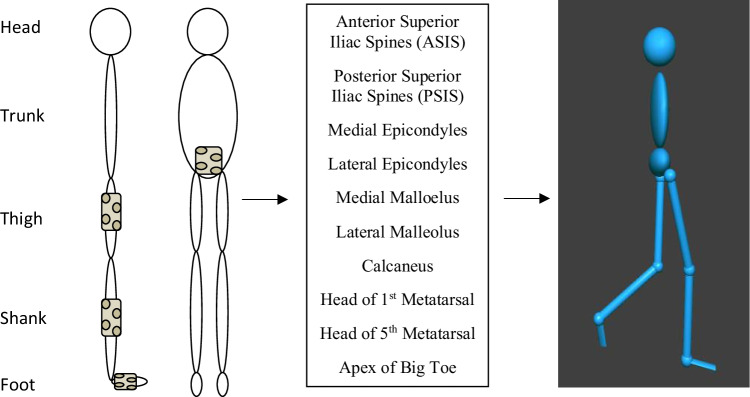
Cluster positions on each segment (left) and anatomical landmarks for calibration (middle) and the avatar after calibration (right)

Each of the testing sites had motion capture systems for measuring biomechanical data (Vicon MX cameras sampling at 100 Hz through Vicon Tracker v3.5.1, Oxford, UK). Data was live-streamed into D-Flow software for the visualisation (Motek Medical, Amsterdam, the Netherlands). Body segments were determined using pointer calibration at 16 pre-determined anatomical landmarks and segment clusters (Strathclyde Cluster Model) which allowed for an avatar to be generated that showed the participants’ pose in real time (Fig. 1) [29, 30]. Joint coordinate systems and kinematics for this model were calculated as per International Society of Biomechanics recommendations [31, 32].

### Stability-based training intervention

The stability-based training programme was derived from standard practice and consistent with the principles of training [33–37]. The programme aimed to improve and challenge ankle joint stability and postural control by incorporating elements relative to activities of daily living, such as single leg standing, change of support, transfer of weight, and coordination. The programme specifically developed balance using four multi-joint and complex exercises (Table 1). All exercises were performed facing forward with hands on iliac crests or relaxed by side. The training was adapted for the inclusion of the visualisation which created a non-immersive, third person perspective virtual environment. The VIS and NO-VIS exercises were very similar to allow a direct comparison as to the effect of the visualisations.

**Table 1:** Training programme including progressions (VIS = visualisation training group, NO-VIS = no visualisation training group)

	Group	Exercise	Description	Intensity	Progression
**Exercise 1**	NO-VIS	Single leg balance with hip flexion	Participant performed single leg balance with contralateral hip flexion. Participant aimed to flex hip and knee to 90°	2 sets × 10 reps 10–12 RPE	Increased intensity: perform 3 sets Sensory manipulation: Vision occluded Wobble board added
	VIS	Single leg balance with hip flexion (Fig. 2a)	Participant performed single leg balance with contralateral hip flexion to target on screen. Range of motion was established before starting the exercise	2 sets × 10 reps 10–12 RPE	Increased intensity: perform 3 sets Sensory manipulation: Wobble board added
**Exercise 2**	NO-VIS	Star Excursion Balance exercise	Participant performed Star Excursion Balance Test (as per outcome assessment)	2 sets × 8 reps 10–12 RPE	Increased intensity: perform 3 sets Sensory manipulation: Vision occluded Wobble board added Cognitive manipulation: Reactivity added
	VIS	Single leg stand and reach (Fig. 2b)	Adapted from Star Excursion Balance Test. Participant reached to targets on screen. Points scored when target touched	2 sets × 8 reps 10–12 RPE	Increased intensity: Perform 3 sets Increase target distance Sensory manipulation: Wobble board added Cognitive manipulation: Spots appear for shorter time
**Exercise 3**	NO-VIS	Lunge with distractive techniques (dual tasking)	Participant performed alternating reverse lunge. One foot stepped posterior to body, and body lowered until the anterior thigh and posterior leg were parallel with the ground [40, 41]	2 sets × 12 reps 10–12 RPE	Increased intensity: perform 3 sets Sensory manipulation: Wobble board added Cognitive manipulation: Arithmetic task
	VIS	Lunge with distractive techniques (dual tasking) (Fig. 2c)	Participant performed alternating reverse lunge	2 sets × 12 reps 10–12 RPE	Increased intensity: perform 3 sets Sensory manipulation: Wobble board added Cognitive manipulation: Stroop test [42]
**Exercise 4**	NO-VIS	Leap-based exercise	Adapted from the Dynamic Leap and Balance Test [43]. Participant leapt to targets following a star pattern on the floor	2 sets × 10 reps 10–12 RPE	Increased intensity: Perform 3 sets Increase target distance Sensory manipulation: Vision occluded Cognitive manipulation: Reactivity added
	VIS	Leap game (Fig. 2d)	The aim was to avoid avatar from being hit by any object on screen by leaping from one leg to the other	2 sets × 30 s 10–12 RPE	Increased intensity: Perform 3 sets Perform for 60 s Sensory and cognitive manipulation: Objects move towards participant at various speeds, distances, and directions

**Fig. 2:**
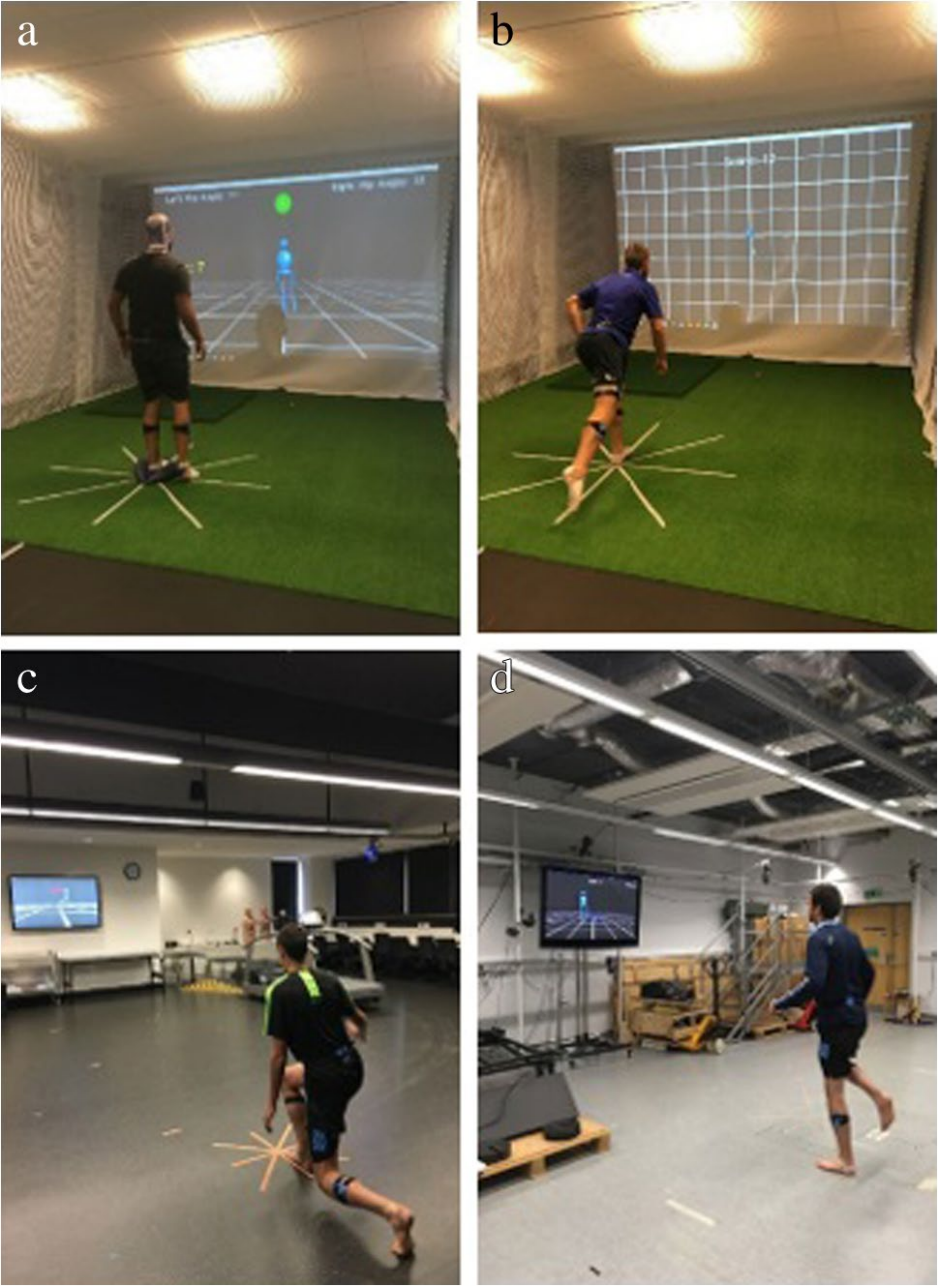
**a**–**d** Examples of stability-based training exercises with visualisation

During training, all participants received verbal feedback using clear and concise cues (i.e. take pelvis straight to floor during lunge, do not cross legs during leaps, control your leg in the single leg balance). The researcher was aware of the potential for performance bias and remained as objective as possible so as not to treat the groups differently during training.

Each exercise was performed at every training session at light-moderate intensity with an expected rate of perceived exertion (RPE) of 10–12 [36, 38, 39]. Exercises were progressed as movement quality for the exercise at the given level was achieved (Table 1) [11–13, 37].

Participants wore comfortable clothing that was suitable for rehabilitative practice, and all testing procedures and training exercises were performed barefoot.

### Outcomes

Feasibility was recorded as rate of recruitment, retention, and adherence to the training intervention. Safety was recorded as the number of adverse events during testing and/or training. Risks included ankle or other musculoskeletal injury from physical activity related to the stability training or falls during training in the laboratory. As in current clinical practice, the training was designed to be progressive enabling participants to stay within their capabilities, and participants were instructed to not perform exercises at levels beyond their ability. Falls risk was mitigated by ensuring the testing, and training space was kept clear of hazards.

Efficacy was reported through the effect of visualisation on dynamic stability, perception of stability, and rehabilitative experience.

At baseline, participants performed the Star Excursion Balance Test (SEBT) and CAIT. At the post-training testing session, the SEBT was reassessed, and the CAIT and Physical Activity Enjoyment Scale (PACES-8) questionnaire was completed.

#### Star Excursion Balance Test

The SEBT is an accepted and reliable method adopted in clinical practice to assess the outcome of stability-related interventions [33, 44–48].

**Fig. 3:**
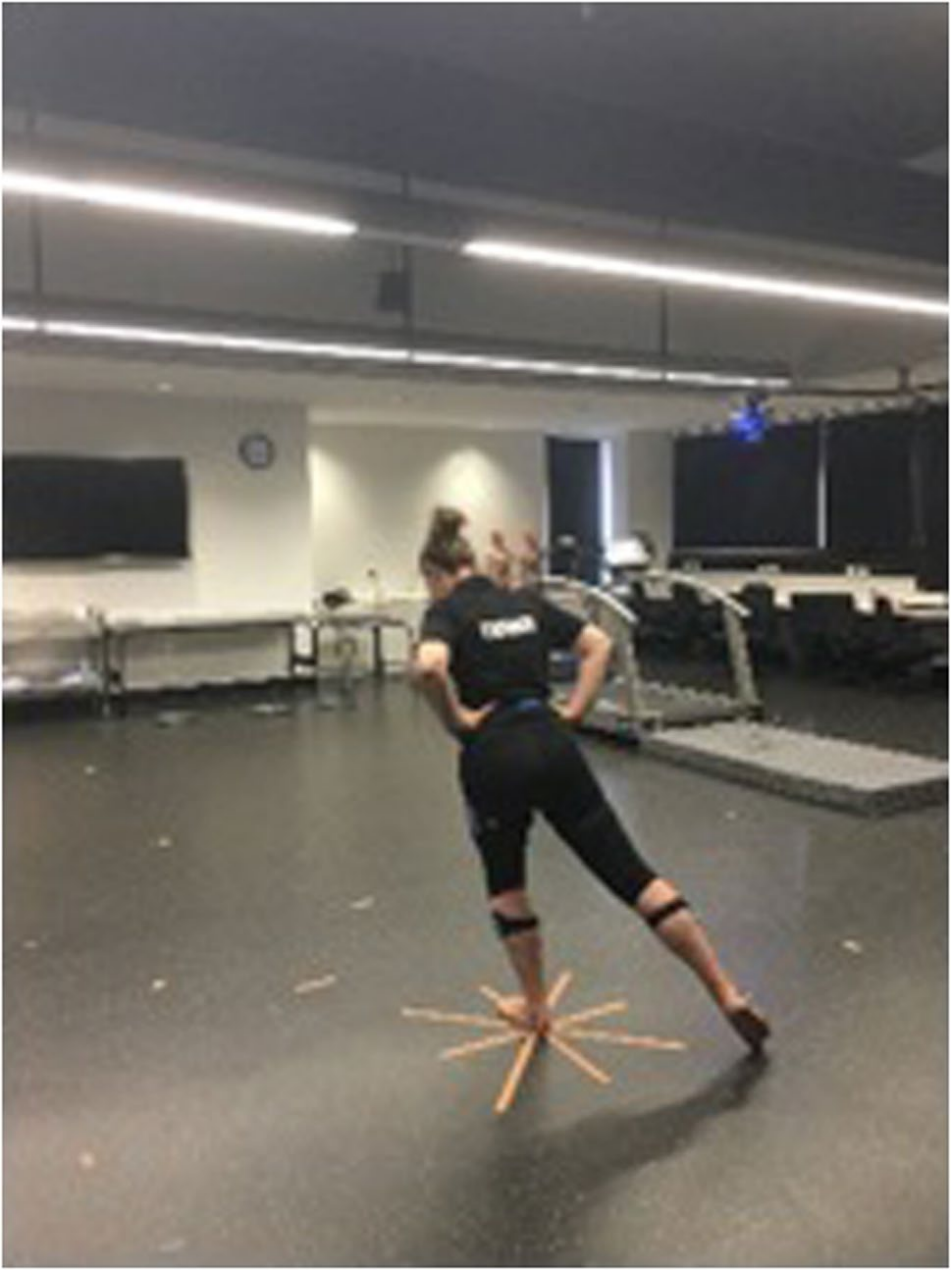
Example of SEBT being performed by participant and tape to guide the reaches

While maintaining unilateral stance, eight maximal reaches were performed (Fig. 3) with each reach normalised to participant leg length and the maximum reach distance for all directions recorded. No weight transfer could occur during each maximal reach [47]. Failure to comply with the verbal instructions meant the trial was discarded and repeated. Up to four practice trials, each side and one test trial, were performed [49, 50].

The change in maximal reach distance from pre- to post-test for the eight reach directions, and average of the eight, was analysed. In cases of bilateral CAI, the ankle perceived as more unstable was analysed. A recent meta-analysis reported a minimal detectable change (MDC) of greater than 8.15% in the PM direction to identify success of a 4-week balance rehabilitation programme [51]. No study has identified the MDC for all eight directions of the SEBT in the CAI population.

#### Cumberland Ankle Instability Tool

The CAIT quantified perception of stability. The minimal detectable change and minimal clinically important difference of the CAIT score is ≥ 3 [52].

#### Physical Activity Enjoyment Scale (PACES-8) questionnaire

The PACES-8 was used to quantify user experience. This measure has been frequently used in rehabilitation research into VR and balance [53–57], however not specifically in a CAI population.

The eight-item questionnaire used a five-point Likert Scale to evaluate the participants’ level of enjoyment — 1 being ‘strongly disagree’ and 5 being ‘strongly agree’, giving a total score out of 40. A high overall score signified high enjoyment of the training.

### Randomisation

Participants were randomly assigned to the NO-VIS or VIS training group using a random number generator [58]. This was generated and allocated by the lead researcher.

### Blinding

Participants and tester remained blind to group allocation until after the pre-training test. Thereafter, neither was blind to the intervention group.

### Statistical analysis

All outcomes relating to the feasibility of the study are descriptively presented. Group data are presented as group means and standard deviation.

Statistical analysis was performed using SPSS (SPSS Statistics: v. 26, IBM, USA). Shapiro–Wilk normality tests were conducted to test for the assumption of normality.

To test if the visualisation improved performance more than stability training alone, the SEBT performance and CAIT were compared using an ANCOVA. The dependent variable was the post-test scores and the independent variable the groups (experimental and control). The pre-test scores acted as a covariate to control for any differences pre-training. The PACES-8 questionnaire was analysed using a 2-sample *t*-test. All tests were analysed at a 0.05 level of significance. The magnitude of the effects was calculated and interpreted using Cohen’s effect size recommendations (*d*), i.e. small: 0.3–0.49; medium: 0.5–0.79; large ≥ 0.8 [59].

## Results

**Fig. 4:**
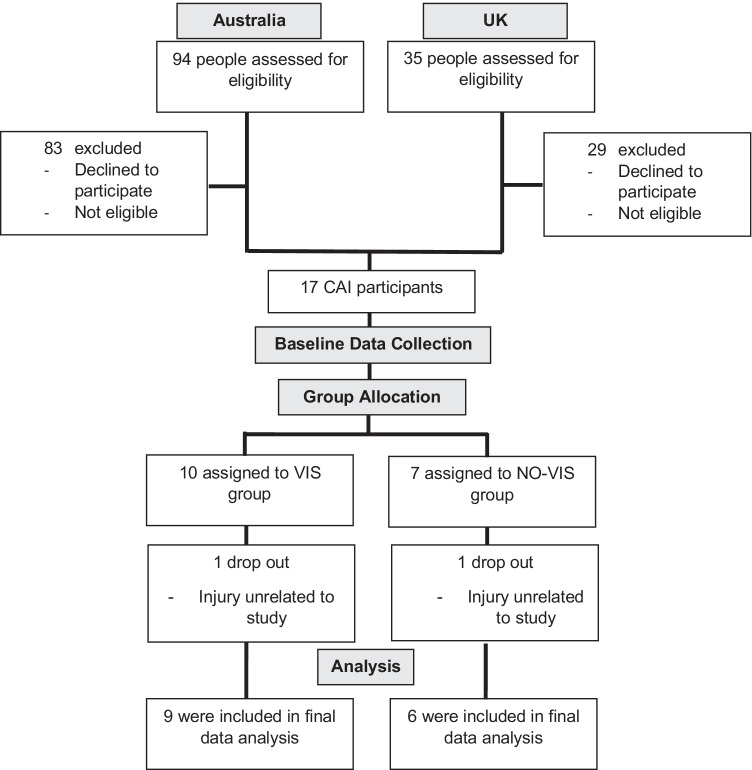
CONSORT flow diagram for participants

From the 129 people assessed for eligibility of inclusion criteria over 4 months in the UK and 1.5 months in Australia, 17 were recruited for the study. This equated to 0.38 and 1.83 participants per week for each site, respectively. The main reason for not participating in the study were people not responding to correspondence after receiving the participant information sheet. Secondary to this was the study requiring a larger time commitment than could be given, no reimbursement for participation, and not meeting the inclusion criteria. This included revealing instability around ankle but no previous ankle injuries, lower limb dislocation, breaks, fractures, and/or surgeries, and recent significant ankle sprains. There were two dropouts during the study — an Achilles injury (VIS group) and an acute injury to the unstable ankle (NO-VIS group). Both were unrelated to the study and prevented continuation of participation. There were no adverse events related to the treatment allocations. The final analysis included 15 participants — an 88% retention rate (Fig. 4).

Attendance at the supervised training sessions was 100% for participants in both the VIS and No-VIS groups. There were no adverse events reported for either training group.

Descriptive statistics are presented in Table 2. There were no between-group differences in population demographics of the VIS and NO-VIS training groups (*p* ≥ 0.1).

**Table 2:** Descriptive statistics (mean ± SD) for all participants

	VIS	NO-VIS
Site (UK/Aus)	4/5	2/4
Age (years)	28 ± 9	29 ± 14
Gender (M/F)	5/4	3/3
Body mass (kg)	78.9 ± 37.3	72.1 ± 9.6
Dominant limb (R/L)	9/0	6/0
Physically active (%)	77.8	100
Competitive athlete (%)	22.2	66.7
Injured ankle (R/L)	4/5	2/4
Number of ankle sprains	3.7 ± 2.8	3.5 ± 1.8
Treatment sought (%)	77.8	66.7
Rehabilitation undertaken (%)	77.8	83.3
Rehabilitation satisfaction (%)	44.4	50

The results of the SEBT showed a greater increase in performance for the VIS group in the posterior-lateral (PL), and lateral (L) directions with large effect (*d* = 1.5–1.8, Table 3). The greatest improvements occurred in the PL and L directions, with improvements of 12.42% and 10.04% in the VIS group, respectively, compared to improvements of 0.33% and 0.56%, respectively, in the No-VIS group. The VIS group also showed a greater improvement in average reach distance (6.74%), compared to the NO-VIS group (0.13%), with a large effect size (*d* = 1.7, *p* = 0.02, Table 3).

**Table 3:** Mean (SD) difference between pre- and post-test results for the SEBT

	VIS		NO-VIS				
Reach direction	Pre	Post	Pre	Post	Mean difference	*d*	*p*
	(% leg length)				(95% confidence intervals)		
Anterior	66.28 (5.6)	67.05 (5.3)	68.13 (5.7)	66.96 (7.0)	1.94 (− 3.24 to 5.84)	0.53	0.54
Anterior-medial	63.77 (5.1)	66.08 (4.4)	66.43 (5.5)	66.43 (6.1)	2.31 (3.24 to 5.84)	0.53	0.54
Medial	58.23 (6.6)	63.63 (6.3)	61.58 (7.8)	62.38 (6.1)	4.61 (− 3.72 to 9.04)	0.67	0.38
Posterior-medial	52.58 (9.7)	61.01 (5.9)	58.40 (9.4)	59.37 (9.2)	7.45 (− 0.68 to 10.89)	1.29	0.08
Posterior	46.29 (11.4)	57.25 (5.7)	53.21 (10.4)	54.69 (7.7)	9.49 (− 1.33 to 11.76)	1.16	0.11
Posterior-lateral	41.09 (8.6)	53.51 (8.0)	49.91 (9.8)	50.24 (7.3)	12.10 (0.72 to 15.77)	1.82	0.03*
Lateral	39.52 (8.7)	49.56 (7.6)	46.28 (9.4)	46.85 (7.5)	9.48 (0.55 to 13.11)	1.56	0.04*
Anterior-lateral	58.97 (11.0)	59.31 (8.6)	61.84 (9.6)	59.23 (6.4)	2.95 (− 5.30 to 8.49)	0.39	0.624
Average	52.97 (6.3)	59.72 (5.3)	58.14 (7.1)	58.27 (5.2)	6.61 (0.86 to 8.78)	1.69	0.02*

*Significant difference between VIS and NO-VIS groups (*p* ≤ 0.05)

**Fig. 5:**
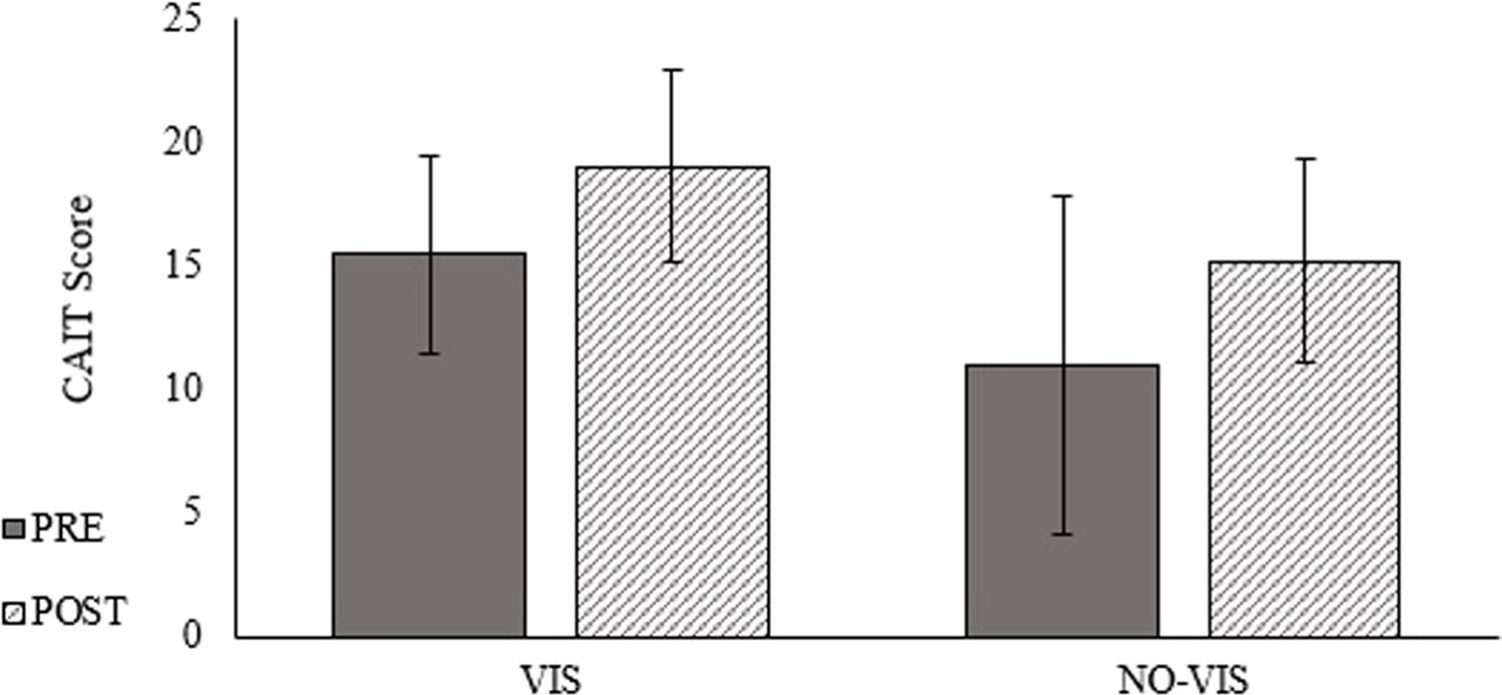
Pre- and post-test CAIT score for the VIS and NO-VIS groups

There were no statistically significant between group mean differences for CAIT (*d* =  − 0.14, *p* = 0.36) (Fig. 5). The CAIT score in each group individually showed clinically meaningful improvements (VIS = 3.6 ± 3.3 points, NO-VIS = 4.2 ± 5.3 points).

**Fig. 6:**
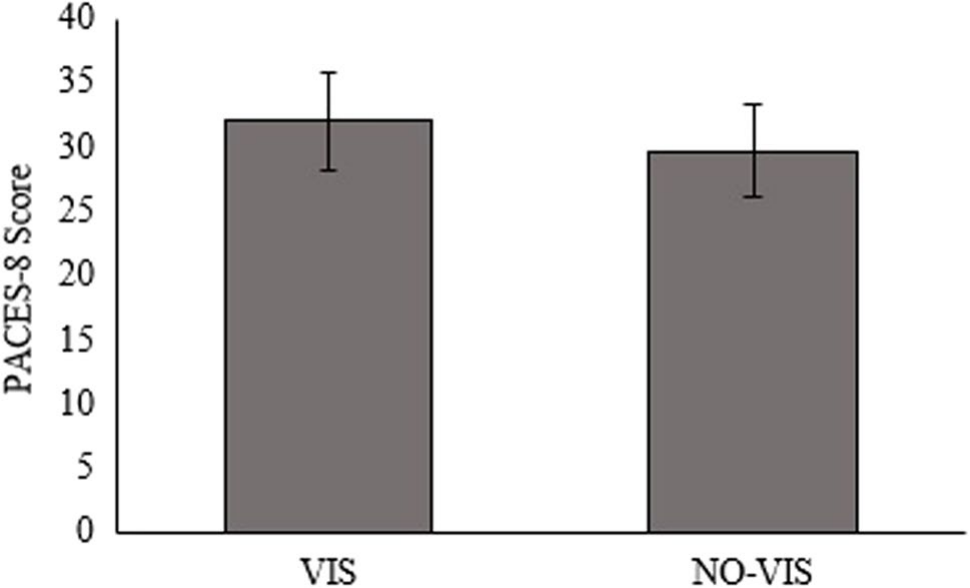
Perception of enjoyment of training for the VIS and NO-VIS groups

For enjoyment, the VIS group did not score significantly higher than the NO-VIS group (*d* = 0.6, *p* = 0.26) (Fig. 6).

## Discussion

This pilot randomised controlled trial assessed the feasibility, safety, and efficacy of stability-based training with visualisation for people with CAI. The study supports the feasibility of the 4-week stability-based training programme with visualisation. The statistically significant between group difference for objective stability, and meaningful change in subjective stability, support the potential efficacy of this training.

The protocol and training intervention using visualisation was shown to be feasible as indicated by the retention and adherence of participants. Safety of the programme was evidenced by no adverse events reported. This suggests that the design and progression of the training was an appropriate intensity, and the visualisations did not present any harm.

### Dynamic stability and perception of stability

The stability-based training with visualisation enhanced performance of the SEBT in the lateral and posterior-lateral directions with large effect. Average reach distance was also improved. The improved reach in the posterior-lateral and lateral directions of the SEBT for the VIS group exceeded the MDC [51, 60]. This was not apparent in the NO-VIS group. Research has suggested reaching posteriorly to the body requires an increased reliance on somatosensory feedback due to lack of visual awareness. This would indicate an increased level of stability is required to maintain position [61]. Considering this, it is promising for potential future implementation of visualisation into rehabilitation practice that the greatest effect of the training occurred in these directions where remaining stable is more challenging. Moreover, to perform maximal reaching in the posterior-lateral direction, there are limited options for the body to position itself, mainly the trunk, above the base of support as opposed to when performing reaches in the medial-oriented directions. Improvements in this direction may suggest that the external focus created by the visualisation both challenged and motivated the participants to find a method to achieve the task that resulted in greater reaching distances when visualisations were removed. Meldrum and colleagues [62] reported that VR training created an external focus which provided a distraction for when practices were repetitive. Augmented feedback and/or an external focus of attention have previously been reported to elicit greater learning effects than an internal focus [14, 15, 63].

Despite improvements in the particular directions discussed above, there were limited effects in the remaining directions. Previous research has highlighted joint range of motion (ROM), and strength has been shown to significantly effect SEBT performance [64–68]. Specifically, ankle dorsiflexion and knee flexion predicts anterior reach distances more than posterior reach directions [64, 65, 69]. Anterior reach distance has also been related to quadriceps muscle strength to control knee flexion during this task [69]. The current programme did not include any specific training to increase ROM or strength. This may in part explain why no improvements in anterior reach distance were observed with the training program. For both the VIS and NO-VIS groups, the improvements in perception of stability following training were of clinical significance [52]. This suggests that the stability-based training interventions both with and without visualisation for people with CAI could be used to improve perception of stability. This supports the previous research that stability-based training is an effective method of rehabilitation [4, 9, 21].

### Rehabilitation experience

Rehabilitation programs require adherence to be effective [21], which is more likely if the programme is enjoyable and the participants are motivated [24]. In this study, participants in both the VIS and NO-VIS groups scored enjoyment highly in the PACES-8 questionnaire. A difference in enjoyment of training between the VIS and NO-VIS groups was hypothesised. Had the NO-VIS training protocol exactly followed that of routine clinical practice, current ankle treatment guidelines, or a programme from a previous study, as opposed to the training programme conducted here, it is believed the difference of enjoyment reported between groups would have been greater. Like the VIS group, the NO-VIS training was designed as a more functional and progressive programme to those seen before, just without the visualisation. Furthermore, the laboratory environment and motion capture equipment may have also influenced the rehabilitative experience. Both groups trained in the laboratory environment, thus, it is possible a difference in enjoyment may have been evident had the control group participated in an unsupervised or at home rehabilitation programme where the environment was not changed and supervision not present. For future work, it would be interesting to examine the enjoyment of a programme completed at home, and not in a clinical or laboratory-based setting.

For both groups, a large majority of participants (≥ 50%) were previously unsatisfied with their rehabilitation. In the current study, participants in both groups reported clinically meaningful improvements in perceived stability, and this may have increased confidence and trust in the program. This would satisfy both the need for competence and relatedness. In turn, this may have increased motivation in both groups, leading to increased effort and engagement [23, 24]. Although this may have been subconscious, this could have resulted in further perceived improvements leading to greater enjoyment from the programme, as it was perceived more successful than previous experiences, and ultimately resulting in only a small difference between VIS and NO-VIS enjoyment scores.

## Study limitations

There are limitations to this study. Firstly, despite the sample population representing a diverse group, the sample size was small, and the randomization of participants led to unequal numbers in each of the groups. Therefore, any inferences from the results should be interpreted with caution. Participant recruitment was impacted by the multi-centre nature of the study. Given the study was conducted internationally across three sites, a new participant recruitment protocol was required each time. However, this is believed to strengthen the study, despite the small sample size, since the protocol was completed in three different locations with no defining impact on retention, adherence, and safety. Given the limited research regarding chronic ankle instability and use of visualisation, the study was designed to assess initial feasibility to establish whether further study would be warranted with a larger sample size, and it is believed this has been achieved. It is important for future work to consider the recruitment rate. Primarily, this should include increased follow up of people who have expressed interest in the study. Further, researchers could consider providing reimbursement to participants. Reducing the time commitment may also be considered, but this may influence efficacy, and the protocol used in this study aimed to represent current clinical practice and research [33–36, 39].

After allocating the participants to the VIS or NO-VIS group following baseline testing, the participants were no longer blinded as to the training group they were part of. Due to the nature of the study this was not possible but is a limitation presenting possible bias. For the testing and training, the lead researcher was the only tester, subjecting the study to further potential bias. However, having only one tester allowed for consistent practice throughout testing and training as would occur in rehabilitative practice.

Due to the nature of the study, supervised laboratory visits were required for study completion. Participants attended all training sessions, but future studies could aim to monitor adherence to visualisation during unsupervised or home-based training scenarios, if facilities and equipment permit. Further to this, future research should use a specific measure for motivation to analyse the specific type of motivation the training may have created relative to the self-determination theory [70] continuum.

This study was sufficiently powered for the SEBT. Based on the results of this study, an estimated sample size of 16 per group would be required for future work to assess enjoyment with visualisations included in the training programme and for the CAIT 62 per group. This was based on the results of the current study at a power of 0.80 and alpha level of 0.05. To be adequately powered for all outcomes, the minimum sample size would be 62, not accounting for dropouts. Future studies could also examine additional or alternative outcome methods of perceived stability, such as the Foot and Ankle Ability Measure [71], as well as conducting short interviews for more in-depth analysis.

## Conclusion

The results of this pilot study support the feasibility and safety of stability-based training with visualisation in those with CAI. We found enhanced performed on the SEBT when training with visualisation which suggests this could be an effective approach to stability-based training. Further investigation using a larger sample and additional subjective measures is needed to thoroughly assess stability and enjoyment when visualisation is incorporate into training.
